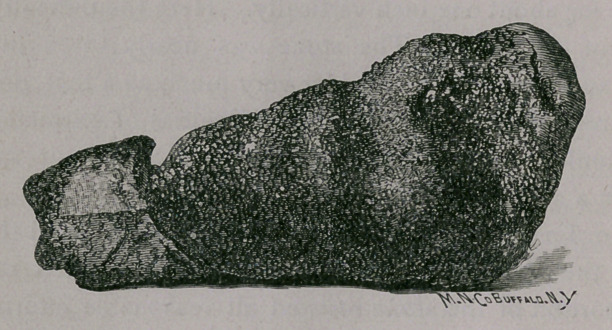# Supra-Pubic Lithotomy*From a paper read before the American Surgical Association, Washington, D. C., May 3, 1884.

**Published:** 1884-09

**Authors:** W. S. Tremaine

**Affiliations:** Major and Surgeon, U. S. A. Fellow of the American Surgical Association. Professor of Surgery, Niagara University. Surgeon-in-Chief of the Buffalo Hospital of the Sisters of Charity, and of the Emergency Hospital, Buffalo, N. Y.


### BUFFALO

### Medical and Surgical Journal.

Vol. XXIV. SEPTEMBER, 1884. No. 2.

### Original Communications.

### Supra-Pubic Lithotomy.*

By W. S. Tremaine, M. D.

Major and Surgeon, U. S. A. Fellow of the American Surgical Association. Professor of Surgery, Niagara University. Surgeon-in-Chief of the Buffalo Hospital of the Sisters of Charity, and of the Emergency Hospital, Buffalo, N. Y.

The *British Medical Journal* of March 12, 1881, in an editorial note, says:

"So rarely is the removal of vesical calculus attempted by the supra-pubic method that though all surgeons are familiarly acquainted with descriptions of the operation, but few can have witnessed its actual performance.

"On the afternoon of Friday, February 25th, Mr. Lister, at King's College Hospital, performed the operation twice. Both patients were males, and in each the strictest antiseptic precautions were observed."

The foregoing extract is my apology for presenting to the association the following case, with remarks thereon:

It is indeed true that the operation is rarely performed, and in the ordinary text books scarcely alluded to, or dismissed as only suitable for stones of very large size, and when the perineal operations are impracticable.

I confess these were my own views in regard to it until the necessity for the operation occurred, as evidenced by the case I now report.

* From a paper read before the American Surgical Association, Washington, D. C., May 3, 1884.

James McH., age 3½, one of thirteen children, born of healthy German parents, delicate looking, not larger than a child two years old, had never walked.

When eighteen months old his mother called a physician on account of retention of urine. This was relieved for the time. For the past two years, the mother states that the condition of the child has been deplorable. The urine passed drop by drop, constant pain was present, violent straining and prolapsus of the rectum frequently occurring.

Five physicians of the city saw him at different times and various remedies were prescribed without effect, as they evidently failed, one and all, to recognize the true state of the case, although, according to the mother's statement, one medical gentleman told her there was probably something the matter with the child's bladder.

Relief was then sought at the Buffalo Medical and Surgical Dispensary, when the calculus was discovered by Drs. Macniel and Davidson, attending physicians.

I was called by these gentlemen to operate. Accordingly, I went to the residence on November 1, 1883, expecting to perform the ordinary lateral operation.

After the child was etherized I examined it for the first time. The stone could be felt with the sound at the vesical neck, but the instrument could not be introduced into the bladder any distance. With the fingers in the rectum the stone gave the impression of being about the size of an ordinary hen's egg; it was immovable.

The distance between the "tuper ischii" was one and one third inches. It was quite evident that to attempt to extract so large a stone through the ordinary perineal section would probably prove impossible, and at any rate result in extreme and serious laceration of the parts.

I had no lithoclast with me, so fragmentation, before extraction, was out of the question, and, as I think the sequel proved, would have been impracticable and dangerous.

Therefore I decided upon the "high operation," an opinion which was concurred in by the medical gentlemen present, Professors Davidson and Lothrop and Doctors Macniel and Potter, the latter my clinical assistant.

About two ounces of a solution of borax was, with difficulty, injected into the bladder.

I then made an incision directly in the median line, commencing over the symphysis pubis and extending upwards for two and one-half inches through skin and superficial fascia; the linea alba sought for and the aponeurosis divided for one and one-half inches vertically.

The peritoneum was seen at the upper extremity of the incision and was carefully pushed up out of the way. An attempt was then made to protrude the anterior wall of the bladder with the point of the sound; this was found to be impossible, as the sound could not be manipulated in the bladder, owing to the large size and impaction of the calculus.

The bladder was then hooked up with a tenaculum and divided for about one inch vertically. Here the difficulty began —that of extraction. The stone was nearly three inches in length and acted to the bladder very much as a tent pole does to an ordinary wall tent, and the difficulties of extraction were very similar to an effort to detach and draw out said pole through a small opening in the wall of the tent.

The ordinary lithotomy forceps were introduced and the upper end of the shaft, so to speak, of the stone broken off. The lower portion of the stone resisted all reasonable efforts at extraction with the forceps.

The finger was then introduced, and the bladder, which was adherent to that portion of the stone occupying the *bas fond* of the viscus, stripped off, and, after cautiously enlarging the opening, the stone was removed. The condition of the bladder was bad from the long-continued cystitis, and it was impracticable to keep a soft catheter through the urethra, as the child would not allow it to remain.

I therefore decided to leave the wound in the bladder open for drainage and introduced a short rubber tube into it.

The upper two-thirds of the incision through the walls of the abdomen was closed with silver sutures; this healed by first intention. The urine escaped continuously through the wound.

Temperature ranged from 101° to 102° for several days. The bladder was daily washed out through the wound with a warm solution of boroglyceride, and occasionally by injection through a soft rubber catheter. Great attention was given to cleanliness. The groin and thighs were kept smeared with benzoated zinc ointment to prevent excoriation from the constant flow of ammoniacal urine over them.

The child gradually improved and the fistula healed in about five weeks.

The stone weighed nine drams, was two and seven-eighths inches in length and one and one-fourth inches at its largest diameter, of peculiar shape, as shown by the accompanying cut, *actual size.*

I believe this to be the largest stone ever extracted from a child of that age, with recovery. At least, in the tables of reported operations, to which I have access, I have been unable, so far, to find any recorded case of recovery after the removal of so large a stone from so young a child.

Notwithstanding the difficulties of this case, from the large size of the stone, I was struck with the ease and safety with which this operation, in an ordinary case, could be performed, and particularly with the simplicity of the apparatus required —an ordinary pocket-case sufficing.

Our distinguished Fellow, Gouley, in his work on diseases of the urinary ogans, enumerates the following accidents of lateral lithotomy:

1st. Failure to reach the groove of the staff and consequent injury of adjacent parts. 2d. Wound of the rectum. 3d. Wound of the interior of the bladder. 4th. Hemorrhage. 5th. Urinary fever. 6th. Infiltration of urine. 7th. Laceration of neck of bladder during extraction. 8th. Cystitis. 9th. Peritonitis. 10th. Pyæmia. To which might be added, 11th, Impotence.

Of these the 1st, 2d, 3d, 4th and 7th may be absolutely eliminated from the supra-pubic operation, a clear gain for this method. *Cœteris paribus.*

The danger from the perineal section, viz., impotence, and not an imaginary one, either, as clearly shown by such authorities as Sir Henry Thompson, Teevan and others, is entirely obviated.

The "high operation" is an old one and is thus spoken of by Cheselden:

"Though the operation (supra-pubic) came into universal discredit, I must declare it as my opinion that it is much better than the old way, to which they all returned, except myself, who would not have left the high way but for the hope I had for a better." *

Endorsed so far back by such eminent authority, why, then, has this operation fallen into such disrepute. Chiefly, I believe, from a superficial examination of statistics, which show a mortality against it.

A closer scrutiny of statistical tables and an impartial investigation of the literature of the subject, a work which has been so thoroughly undertaken by Dulles (*American Journal of Medical Science*, July, 1875), and more recently by Helmuth, ("Supra-Pubic Lithotomy," by William Tod Helmuth, New

* A Treatise on High Operation for Stone, with seventeen copper plates. London, 1723.

York, 1882), will convince any candid mind that the operation has not received the consideration due to it, and that it should take its place among the cutting operations for stone as of equal, if not greater, safety in regard to mortality, and superior as to unpleasant consequences, such as impotence, etc.

Given, a foreign body to be removed from a viscus, the most direct and safest route to reach it would naturally suggest itself; that the "high operation" is the most *direct*, goes without saying; that it is anatomically the safest, needs no demonstration; that, apart from mortality, which, under like conditions, is in favor of epicystotomy, it is safer as regards after consequences, and is *the* method—the natural, safe, direct route to the interior of the bladder, when section is necessary, and the burden of proof is on the advocates of perineal section, to show wherein there are any advantages by the circuitous, dangerous, dark route over the safer and easier method by epicystotomy.

The supra-pubic operation is relatively easy—it gives more room than the perineal section, and enables the operator to see just what he is doing.

### ADDENDUM.

Since the above was written, I have again performed supra-pubic lithotomy, or, more correctly speaking, supra-pubic cystotomy, or epicystotomy. We do not cut the stone, but, rather, the bladder.

Conrad F., aged 30, was sent to me by Dr. Cole, of Middleport, N. Y., with a history of ten years' suffering from symptoms of stone in the bladder.

Examination with the sound showed the presence of a large calculus.

The patient was etherized, and an attempt at litholopaxy made, when it was found that the stone was very large and firmly lodged above the symphisis pubis, forming, as it were, a lining to the anterior wall of the bladder. All attempts to dislodge and grasp the calculus were unsuccessful, but what seemed to be a separate stone was crushed, and the detritus, to the amount of one ounce, was removed by Bigelow's improved evacuator.

This operation set up a violent cystitis; for a week or ten days fragments of stone passed the urethra. The man suffered greatly, and at times his condition seemed critical.

He gradually improved, and on June 4th, at the amphitheatre of the Buffalo Hospital of the Sisters of Charity, in presence of the medical class and Professors Cronyn, Davidson and Heath, with the assistance of Drs. Potter, Clark and Mickle, I operated, by epicystotomy.

About ten ounces of a warm solution of borax and glycerine was injected into the bladder.

An incision about three inches long was made in the median line, just above the pubes. The very slight bleeding from a few twigs in the skin was stopped by hemostatic forceps.

A sound was then introduced through the urethra, the anterior wall of the bladder pushed up, and seized and held by a tenaculum. An opening about one inch in length was made into the bladder, and a number of fragments of stone extracted by using the fore-finger and thumb (more intelligent and safer forceps than any yet devised). These fragments varied in size from one to two inches in diameter, and weighed, together, a little in excess of three ounces, including the nucleus, which was nearly round, seven-eighths of an inch in its longest diameter, and weighed fifty grains. The bladder and wound were washed out with a solution of mercuric bichloride, one part to fifteen hundred of water, the opening closed with four catgut sutures, the external wound united by silver sutures, a small drainage tube left in the lower angle of the wound, a pad of "sublimated gauze" applied, and over all a large gauze bag of sublimated peat. A soft rubber catheter was retained in the urethra. For a few days there was a slight flow of urine from the wound, but the patient recovered in ten days without a single uncomfortable symptom, and three weeks after the operation stated that he "never felt better in his life."

I have been thus minute in describing the details of this case because I believe it is the careful attention to these so-called minor details that contributes largely to the success in modern surgery.

It has been suggested, as one of the objections to the operation, that the anæsthetic might cause vomiting, and so endanger the wounding of the peritoneum.

In this case there was a constant convulsive action of the abdominal muscles. To guard against any trouble from this source, Dr. Potter kept his finger covered with an antiseptic towel pressed on the upper angle of the wound.

I think, danger of wounding the peritoneum and descent of the intestines is purely theoretical, at any rate, not likely to happen in ordinarily skillful hands.

At a meeting of the Leeds and West Riding Medico-Chirurgical Society, May 2, 1884, Mr. T. Pridgin Teale related a case of rupture of the bladder, which he treated by abdominal section. Mr. Mayo Robinson also related a case of like nature, in which he had also performed abdominal section. Mr. McGill remarked, "that the case was of great interest as proving that healthy urine might be effused into the abdominal cavity without inducing peritonitis, a fact which had been doubted by some surgeons."

I neglected to state above that the bladder was daily washed out through the catheter with a solution of iodoform and starch.

As regards the relative merits of epicystotomy and litholopaxy, recently discussed by Dittel, in properly selected cases, and in *experienced and skillful* hands, the former will probably maintain its present position, but my own opinion is that suprapubic cystotomy will prove by far the *safer* operation in the hands of the average surgeon.

With the experience already gained in the *technique* of the operation, I hope, in future cases, by careful suturing of the bladder with a modification of the suture of Lembert, and using fine catgut, to obtain immediate union of the bladder, although, in many cases of long standing, and where chronic cystitis exists, it is probably more desirable that a fistula be established and kept open for a time, in order to drain and rest the diseased bladder.

In operations of this class it seems to me desirable to avoid, as far as practicable, the *genital* organs. By so doing there will be less reflex disturbance of the nervous system.

Epicystotomy accomplishes this—an argument in its favor that cannot be applied to any other operation for the relief of stone, either of the *crushing* or *cutting* variety.

BUFFALO, N. Y., July 3, 1884.

## Figures and Tables

**Figure f1:**